# Gold nanoparticle formation as an indicator of enzymatic methods: colorimetric l-phenylalanine determination

**DOI:** 10.1007/s00216-022-03900-3

**Published:** 2022-01-21

**Authors:** Alba Martín-Barreiro, Susana de Marcos, Javier Galbán

**Affiliations:** Nanosensors and Bioanalytical Systems (N&SB), Analytical Chemistry Department, Faculty of Sciences, Instituto de Nanociencia y Materiales de Aragón (INMA), CSIC-University of Zaragoza, 50009 Zaragoza, Spain

**Keywords:** l-Phenylalanine, Gold nanoparticles, Enzymatic determination, Colorimetric, Enzymatic formation

## Abstract

An enzymatic-colorimetric method has been developed based on the reaction between l-phenylalanine (l-Phe) and the l-amino acid oxidase (LAAO) in the presence of Au(III), which has led to the formation of gold nanoparticles. The intensity of the localized surface plasmon resonance (LSPR) band of the generated nanoparticles (550 nm) can be related to the concentration of l-Phe in the sample. The mechanism of the LAAO-l-Phe enzyme reaction in the presence of Au(III) has been studied through the evaluation and optimization of experimental conditions. These studies have reinforced the hypothesis that the catalytic center of the enzyme helps the Au(III) reduction and, thanks to the protein, the Au^0^ form is stabilized as gold nanoparticles (AuNPs). In the calibration study, a sigmoidal relationship between the concentration of the substrate and the LSPR of the nanoparticles was observed. The linearization of the signal has allowed the determination of l-Phe in the range from 17 to 500 µM with an RSD% (150 μM) of 4.8% (*n* = 3). The method is free of other amino acid interference normally found in blood plasma. These highly competitive results open the possibility of further development of a rapid method for l-Phe determination based on colorimetry.

**Graphical abstract:**

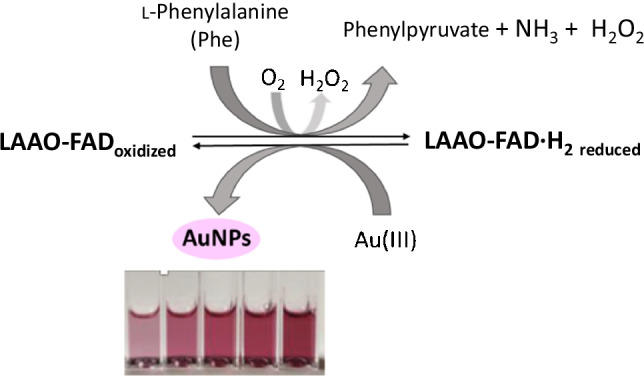

**Supplementary Information:**

The online version contains supplementary material available at 10.1007/s00216-022-03900-3.

## Introduction

Colorimetric biosensors stand out for possessing several key factors such as simplicity and a rapid response through visual detection, without the need for instruments for such detection, and the possibility of quantification using simple equipment. However, current strategies used for colorimetric determinations based on oxidase reactions need coupling with an enzyme HRP-based enzyme indicator reaction, which catalyzes the conversion of chromogenic substrates (for example, TMB or ABTS) in colored products after the oxidation process [1, 2]. The use of this type of colorant presents problems such as their low stability, complex redox behavior, and the possibility of their partial oxidation by the O_2_ present in the air. In addition, reducing species in the sample can interfere with the oxidation of these dyes or they can react with the analyte product [3, 4]. The nanotechnology applied in this type of biosensors through the use of nanoparticle plasmon resonance allows in many cases the sensitive and simplest detection of biomolecules, avoiding these kinds of problems.

Gold nanomaterials (AuNMs) have attracted the attention of the scientific community due to their unique electrical, catalytic, and optical properties dependent on the nanometric dimension and representing an important field in biological and chemical sensing, catalysis, and medicine [5, 6]. The study of the synthesis mechanisms and factors that affect the variability of the properties of these AuNMs has enabled them to be adjusted according to different needs and applications.

The incorporation of AuNMs as labels for the recognition of biomolecules represents an improvement in sensitivity, stability, and biocompatibility compared to conventional colorimetric and fluorometric labeling biosensors.

One of the most common options for the development of nanobiosensors is the prior synthesis of nanoparticles, and in the case of gold nanomaterials, AuNPs or AuNCs are used as substitutes for the chromogenic reagent [7] or fluorophore [8], respectively. Three fundamental synthesis ideas are explored:

1. Use of the intrinsic reducing capacities of proteins: some proteins are capable of generating Au nanostructures due to the reducing capacity of some amino acids [9] and in turn act as stabilizing ligands in the synthesis of nanoparticles or gold nanoclusters [10, 11]).
2. Use of an enzymatic reaction for the generation of AuNPs: It has been observed that in some enzymatic oxidation reactions in which O_2_ acts as an oxidant, the formation of AuNPs is possible in the presence of Au(III). During the enzymatic reaction, Au^0^ is formed, which is stabilized as AuNPs thanks to the protein part of the enzyme, acting in turn as a ligand. The specific mechanism of the formation of Au^0^ depends on the enzymatic reaction, and it is necessary to elucidate it, although it seems to be related (at least in part) to the regeneration of the active center of the enzyme. However, in all cases, the spectroscopic properties of these nanoparticles depend on the substrate concentration [12].
3. Etching or growth of previously formed AuNPs: some authors also indicate that the H_2_O_2_ by-product of some enzymatic reactions (proportional to the substrate) is capable of increasing the growth of previously formed AuNPs [13, 14] or that other products of enzymatic reactions are capable of generating the same effect [15]. However, other authors report the opposite effect, showing that the presence of H_2_O_2_ and/or by-products of the enzymatic reaction in some cases produces a reduction in the size (etching) of the nanoparticles [16, 17].

In the present study, the design of AuNMs was evaluated by means of their directed and specific synthesis with enzymes, exploring the possibilities of a combination of the three different synthetic-sensory enzymatic effects mentioned above.

The flavoenzyme l-amino acid oxidase (LAAO) was selected as a model, an oxidoreductase that catalyzes the oxidative deamination of l-amino acids (l-phenylalanine being one of highest affinity) to keto acids releasing ammonia and hydrogen peroxide. Quantitative determination of l-Phe in physiological fluids is crucial in the diagnosis and therapy of disorders of phenylalanine catabolism, such as phenylketonuria (PKU), a genetic disorder characterized by a deficiency in the liver of the hepatic enzyme phenylalanine hydroxylase that catalyzes the conversion of phenylalanine into tyrosine, leading to an excessive l-Phe accumulation in the serum (> 120 μM) [18]. These levels are most commonly monitored by chromatography methods (LOD 1 μM) [19] or bacteriological inhibition assays (BIA) such as the Guthrie methodology (LOD 120 μM) [20, 21]. These methods give very good results but are time consuming, so faster response methods are desirable.

Finally, a simple enzymatic-colorimetric method has been developed based on the reaction between l-Phe and the enzyme LAAO in the presence of Au(III), which leads to the formation of gold nanoparticles whose absorbance can be related to the concentration of l-Phe in the sample.

## Materials and methods

### Synthesis and characterization of AuNMs stabilized by LAAO

The in situ synthesis of AuNMs was performed using only the reducing residues of the enzyme LAAO (EC 1.4.3.2) derived from *Crotalus adamanteus* snake venom (A9253, Sigma-Aldrich) with different molar excesses of a gold salt sodium tetrachloraurate (III) dihydrate, AuCl_4_Na·2H_2_O (99.8% Au, Stream Chemicals). The final solutions were evaluated by fluorescence using a Varian Cary Eclipse luminometer and by absorption spectroscopy using an Agilent 8453A UV–vis single-beam spectrophotometer.

The final optimized synthesis was carried out by mixing 500 μL of an aqueous solution of L-AAO 4.8 · 10^−5^ M previously tempered at 37 °C and 500 µL of an aqueous solution of AuCl_4_Na.2H_2_O 1 · 10^−2^ M also tempered and stirred for 15 min at 37 °C. Then, 50 µL of a 1 M NaOH aqueous solution was added to adjust the pH to 12, and the mixture was incubated at 37 °C for 24 h, monitoring its fluorescence and absorbance during the reaction time.

The size and morphology of the nanoparticles were studied by STEM with Tecnai F30 (FEI) equipment (at the Laboratory of Advanced Microscopies, LMA, Zaragoza).

Also, in order to study the properties of the generated nanoparticles, gel agarose electrophoresis separation was performed. 2.5% agarose gel (SeaKem LE Agarose, Lonza) was prepared in 0.5 × TBE electrophoresis buffer (Tris, Borate, OmniPur EDTA). This solution was heated in the microwave until a homogeneous solution was achieved. The resulting mixture was poured onto the electrophoresis mold, and the comb was placed to create the wells. After 30 min, the solidified gel was immersed in the horizontal electrophoresis cell filled with 0.5 × TBE buffer, the comb was removed, and the samples were injected into the wells using a micropipette, 50 μL of the sample and 5 μL of 25% glycerol in 0.5 × TBE to give the sample density. Electrophoresis was carried out at 120 V for 20 min. After electrophoresis, the fraction of solution that remained in the well was collected and its fluorescence characterized by performing a 3D spectrum with a Perkin Elmer LS 55 fluorescence spectrophotometer and comparing with the sample before electrophoresis. Furthermore, the observation of these gels by means of a UV lamp (ChemiDoc XRS + System with Image Lab, BIO-RAD) allowed the fluorescent characterization of the sample inside the gel.

### Synthesis of AuNMs stabilized by LAAO in the presence of l-Phe

Cuvette measurements were carried out at a constant temperature of 37 °C. In all cases, 1380 µL of LAAO 3.0 · 10^−6^ M L in a 0.1 M phosphate buffer solution at pH 6.5 was added to the cuvette; then 100 µL of the corresponding concentration of l-Phe was added. After 20 min of reaction, 20 µL of the 5 · 10^−2^ M Au(III) solution was added. The spectra (from 350 to 700 nm) were performed on a Specord 210 Plus spectrophotometer thermostatically controlled at 37 °C. After 4 h at 37 °C, the absorption value at 550 nm was recorded. The reaction kinetics was improved with the addition of 100 total U of catalase. In this case, the absorbance value at 550 nm after the stabilization of the signal (3 h) was used as analytical signal. The size and morphology of these final nanoparticles obtained were studied by STEM with Tecnai F30 (FEI) equipment.

Using the same procedure as described above, an interference study of the other l-amino acids most abundant in blood plasma was performed. The amino acid concentrations used for this study were those considered normal in plasma [22]: l-glutamine 550 µM, l-valine 230 µM, l-serine 120 µM, and l-cysteine 110 µM. These l-amino acid concentrations were added to the cuvette with the enzyme, also together with l-Phe 800 µM (a concentration which corresponds to the interval in which the disease is active), and finally, the gold salt was added. The spectra of the different mixtures were performed on a Specord 210 Plus spectrophotometer thermostatically controlled at 37 °C.

### l-Phe quantification in human blood plasma

The evaluation of the method for the determination of l-Phe in blood plasma samples (Normal, Pooled Human Donors from Dismed, Spain) was carried out using small-volume cuvettes (*V*_final_: 600 μL) according to the following optimized protocol: 3.0 · 10^−6^ M of the LAAO enzyme and 100 total U of catalase were added to a 1/2.5 plasma dilution in 0.1 M phosphate buffer pH 6.5, then 100 µL of l-Phe at the necessary concentration was added. After 20 min of reaction, 8 µL of 5 · 10^−2^ M Au(III) was added and left to react at 37 °C. The absorption measurements of the cuvettes at 550 nm were made using the Specord 210 Plus spectrophotometer after the stabilization of the signal (3 h).

## Results

### Study of the structure and amino acid residues of the LAAO enzyme

A small number of proteins have been explored to synthesize AuNMs [11], only in the presence of a basic medium and a gold salt, using the protein as a capping and reducing agent at the same time. The proteins are lysozyme [23], human transferrin [24], trypsin [25], pepsin [26], peroxidase [27], and bovine serum albumin (BSA) [9], the AuNCs@BSA obtained being one of the most widely used AuNC syntheses and standing out for its high photostability and yield. The influence of the contents and amino acid sequences of these proteins affects the optical properties of the synthesized AuNCs, as well as the secondary formation of larger AuNPs with surface plasmon. However, in all cases, established mechanisms are observed as the basis for this synthesis type:

- *Amino groups of positively charged amino acids* such as arginine (arg), lysine (lys), and histidine (his) are responsible for the coordination of AuCl_4_^−^ ions through electrostatic interactions [28]. The presence of these groups determines the amount of Au incorporated in the enzyme.
- *The reducing residues* tyrosine (tyr) and tryptophan (trp) reduce Au(III) ions at pHs above 10 and influence the degree of reaction to form AuNPs or AuNCs [29].
- *Cysteine residues* (cys) stabilize the formation of AuNMs due to the thiol-gold interaction. Disulfide bonds are hidden within the secondary structure of the protein and are inaccessible in the pH range of 5–7.3. These bonds gradually become available at higher pHs [30].

Based on these hypotheses, the effects of protein size and amino acid content on the formation of AuNMs and their resulting optical properties have been studied [11]. The most important conclusions are:

- The balance between residues containing amine and the reducing residues tyrosine and tryptophan is critical for the formation of AuNCs. Proteins with few amine residues and a greater amount of tyrosine/tryptophan are not capable of producing nanoclusters and generate larger AuNPs.
- The cysteine content is also critical. A lower cysteine content causes AuNCs with emissions at lower wavelengths compared to the fluorescence of AuNCs obtained from proteins with high cysteine content at longer wavelengths.
- The stability and photostability of these AuNMs largely depend on the size of the protein (on which the number of amino acids also depends). Larger proteins lead to AuNMs with better and more stable coatings.

To know the possibilities of the LAAO enzyme in the synthesis of AuNMs, its structure and amino acid residues were studied. The LAAO variant used from *Crotalus adamanteus* is a dimeric glycoprotein with a similar structure to *Crotalus rhodostoma* (2iid.pdb), whose amino acid sequence coincidence is 83% and whose sizes are similar, 117.4 kDa for the adamanteus species and 132 kDa for rhodostoma. Using the 2iid.pdb file and the molecular visualization program Pymol ‘Build’ (Molecular Graphics System, Pymol), the 3D structure of the enzyme was studied and the amino acid residues of interest were counted and designated in different colors (Figure S1.1, ESM1). These characteristics were compared with those obtained for the BSA which is commonly used in the synthesis of AuNCs. Thus, as can be seen in Figure S1.1, the LAAO enzyme has an amino acid composition for the formation of AuNCs even more favorable than that of BSA, which justifies the good possibilities of LAAO for this synthesis.

### AuNMs stabilized by LAAO

The key factor in this synthesis is the concentrations of the gold salt and the enzyme to be added. Different enzyme:Au(III) ratios (1:1, 1:10, 1:50, 1:100, 1:150) were tested using a 4.8 · 10^−5^ M LAAO concentration. The fluorescence spectra of the different solutions showed an emission maximum around 620 nm with excitation at 335 nm only for the two highest gold concentrations (Fig S2.1, ESM2). The results obtained for the 1:100 and 1:150 ratios were practically the same, so the 1:100 ratio was selected as optimal in order to avoid adding more acid medium to the enzyme. Using this ratio, various enzyme concentrations were also evaluated (7.4 · 10^−7^, 3.0 · 10^−6^, 1.2 · 10^−5^, 4.8 · 10^−5^ M), observing the formation of the nanoparticles only from the concentration of LAAO 1.2 · 10^−5^ M and the highest yield for 4.8 · 10^−5^ M LAAO. Higher concentrations of enzyme do not allow its complete solubility.

The AuNMs synthesized with the optimized protocol (Figure S2.2, ESM2) were characterized by STEM. Figure S2.3A shows the STEM images obtained where two clear populations of spherical particles are observed, whose size distributions are indicated in Figure S2.3B. This histogram shows a population of small particles (8.3 ± 2.7 nm) and another population of large nanoparticles (27.1 ± 6.9 nm), which tend to clump together and form groups of about 7 nanoparticles surrounded by organic matter.

The optical properties of the final synthesis were evaluated by fluorescence and absorption. Figure S2.4A shows the absorption spectrum of the AuNPs with a maximum at 530 nm, coinciding with the reddish color observed in the final solution. Figure S2.4B shows a 3D fluorescence spectrum; three fluorescence peaks are located, FAD fluorescence (*λ*_ext_ = 375 and 450 nm; *λ*_em_ = 520 nm) and AuNM fluorescence (*λ*_ext_ = 350 nm–*λ*_em_ = 630 nm and *λ*_ext_ = 330 nm–*λ*_em_ = 420 nm); the latter was initially assigned to the AuNPs that give the solution a reddish color due to their absorption at 530 nm. This hypothesis is supported by diverse bibliography that defines the existence of nanoparticles with both plasmon and fluorescence in this area of the spectrum [31]. In addition, in order to experimentally assign the observed optical properties to one of the STEM populations, the separation of these nanoparticles was carried out by agarose gel electrophoresis, confirming the assigned optical properties (ESM2, Figure S2.5 and Figure S2.6).

### AuNMs stabilized by LAAO in the presence of l-Phe

The previous results showed the feasibility of using the LAAO enzyme in the synthesis of AuNMs. Therefore, the possibility was studied of using this property during the enzymatic reaction with l-Phe, posing a second hypothesis: in addition to involving the amino acid residues of the LAAO enzyme, trying to use the redox properties of the enzyme during the enzymatic reaction to promote the synthesis of AuNMs. This methodology is intended to enable the nanoparticles to be synthesized in situ with the analyte l-Phe and their optical properties, such as absorbance, to be directly related to the substrate concentration. The nanoparticles resulting from this synthesis did not show fluorescence, and their STEM images showed a single population of small nanoparticles of 6.4 ± 0.7 nm, to which the plasmon observed around 550 nm is attributed. Figure S3.1A shows a clear population of spherical particles, whose size distribution is indicated in Figure S3.1B.

#### Mechanism of the LAAO-l-Phe enzyme reaction in the presence of Au(III)

**Fig. 1:**
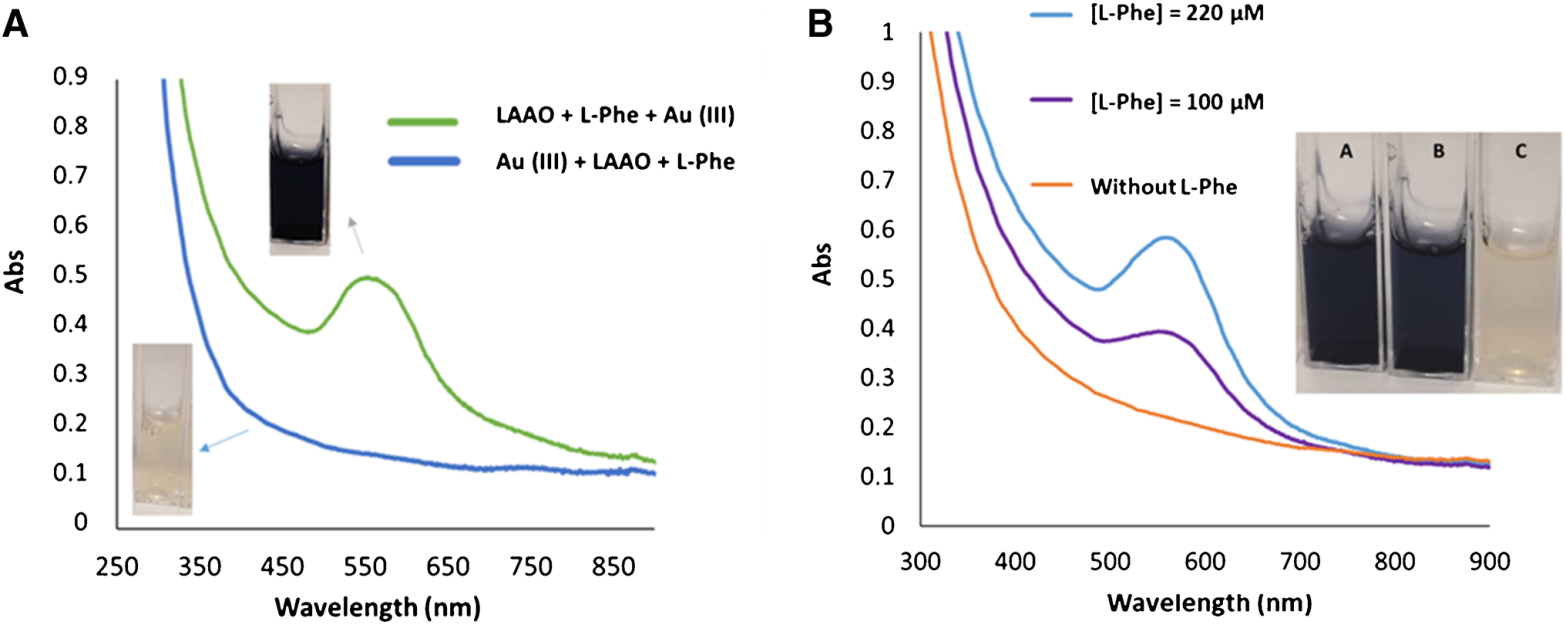
**A** Molecular absorption spectra obtained (20 h) after the reaction of 1.8 · 10^−6^ M of LAAO and 1 · 10^−3^ M Au(III) in the presence of 220 μM phenylalanine in TRIS buffer solution pH 6.5 0.1 M using the orders of addition indicated. **B** Molecular absorption spectra obtained at the end of the reaction of LAAO and Au(III) in the presence and in the absence of phenylalanine. Experimental conditions: [Au(III)] = 1 · 10^−3^ M, [LAAO] = 300 mM, TRIS buffer solution pH 6.5 0.1 M, with blue line [l-Phe] = 220 μM, violet line [l-Phe] = 100 μM, and yellow line without l-Phe; the spectra were obtained after 20 h of reaction

First, two options were studied regarding the order of addition of the reagents: (1) LAAO + l-Phe + Au(III) or (2) Au(III) + LAAO + l-Phe. In both cases, the reaction was carried out for 20 h. Figure 1A shows the results obtained for both cases, in which the need for the enzymatic reaction prior to the addition of gold is observed; otherwise, the appearance of plasmon around 570 nm is not observed.

The intrinsic reducing capacity of LAAO was described in “Study of the structure and amino acid residues of the LAAO enzyme” showing that these proteins are capable of generating AuNMs through the participation of some of their amino acid residues. This requires an excess of Au(III) (~ 5 · 10^−3^ M) and a high concentration of the enzyme LAAO (4.8 · 10^−5^ M). However, in this case, the proposed methodology does not require such high amounts of gold and enzyme, since it is intended that in the absence of the phenylalanine analyte, the formation of AuNPs is not observed. Figure 1B shows an initial experiment in which the reducing capacities of LAAO are evaluated in the presence and absence of the analyte using low concentrations of gold and enzyme. In this way, it can be observed that in the absence of phenylalanine, there is no formation of any type of nanostructure, and no optical property (plasmon or fluorescence) is observed in the solution. In contrast, in the presence of the phenylalanine substrate, the existence of nanoparticles with surface plasmon is observed. In Fig. 1B, it is also possible to observe changes in the intensity of the plasmon band as a function of the concentration of the l-Phe present, due to the fact that the formation kinetics of AuNPs increases when the concentration of l-Phe increases.

**Scheme 1:**
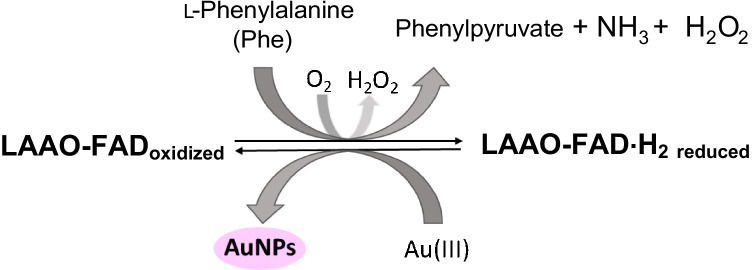
Scheme of the reaction of the LAAO oxidoreductase enzyme with its analyte l-Phe in the presence of Au(III)

In this regard, it is important to point out that the FAD center of LAAO catalyzes the oxidative deamination of l-Phe. During the reducing half reaction, the amino acid is oxidized to the imino acid with the consequent reduction of the cofactor FAD. The imino acid oxidation product undergoes non-enzymatic hydrolysis to give the respective α-keto acid and ammonia. Finally, an oxidative half reaction completes the catalytic cycle by re-oxidizing the FAD with molecular oxygen and producing hydrogen peroxide. The possibility that metal ions may act as substrates for enzymes has recently been verified [32], which reinforces the hypothesis that the active center of the enzyme may be responsible for the formation of AuNPs. If so, after the LAAO-l-Phe enzymatic reaction, the catalytic center of the reduced enzyme would be regenerated by the Au(III) salt, which would be reduced to Au^0^, producing the reoxidation of the active center of the enzyme. Thanks to the protein present, the Au^0^ form is stabilized as AuNPs (Scheme 1).

#### Synthesis optimization of AuNMs stabilized by LAAO in the presence of l-Phe

Several experimental factors were studied in this methodology following the procedures described in experimental section “Synthesis of AuNMs stabilized by LAAO in the presence of L-Phe.” The most important are:

- Nature of the buffer solution and its pH: affects the redox power of Au(III) and also the kinetics of the enzymatic reaction and the stability of the AuNPs.
- Amount of LAAO enzyme: affects the wavelength shift, the stability of the AuNPs formed, and the intensity of the plasmon, which limits the sensitivity of the method.
- Au(III) concentration: higher concentrations generate a greater quantity of AuNPs or a larger size, also affecting the wavelength shift.
- LAAO-Phe enzymatic reaction time before the addition of gold: this is the conversion time of l-Phe to its phenylpyruvate product. A longer enzymatic reaction time before adding the gold implies a higher conversion of the substrate and therefore a greater reduction of the enzyme. This time affects the speed of formation of the AuNPs.

First, the kinetics of formation of these AuNPs in phosphate buffer solution and TRIS buffer were evaluated. Figure S3.2 shows the evolution of the absorbance at the maximum (550 nm) during the synthesis time of AuNPs starting from time 0, which we consider as the moment when gold is added to the solution. Through these results, we can confirm that the formation kinetics of AuNPs is disadvantaged by the presence of TRIS in the medium compared to the existence of phosphate ions. Feng Chen et al. [33] explain the unusual acid–base and redox activity shown by various organic compounds such as Tris (hydroxymethyl) aminomethane, or 2-amino-2- (hydroxymethyl) -1,3-propanediol, capable of coordinating with various ion metals, such as Au(III). Thus, the hypothesis to justify this kinetic behavior has its origin in the complexing of Au(III) by TRIS, which in addition to leaving the reagent in a less accessible form for the enzyme, will lead to the potential decrease of the Au(III)/Au^0^ and, therefore, of the oxidizing capacity of the reagent.

Using buffer phosphates 0.1 M, a sequential optimization was then carried out at a constant temperature of 37 °C, studying the experimental factors mentioned. The results of the different optimizations for the development of this methodology are shown in Figure S3.3, and its results are summarized as follows: using 0.1 M phosphate buffer, pH 6.5 greatly favors the enzymatic reaction over pH 7; the concentration of Au(III) less than or equal to 5 · 10^−4^ M generates small amounts of nanoparticles; however, from 6 · 10^−4^ M, the synthesis yield increases, observing its maximum at 7 · 10^−4^ M. Regarding the amount of enzyme LAAO, the results obtained show that the use of 3.0 · 10^−6^ M LAAO provides greater intensity, without producing a wavelength shift and therefore without aggregation of AuNPs, as is the case with higher concentrations. Finally, the time of the LAAO-l-Phe reaction was evaluated before the addition of gold, showing that the addition of Au(III) after 20 min makes it possible to complete the enzymatic reaction to a greater extent and obtain a maximum rate of AuNP formation.

#### Calibration study of the method for the determination of l-Phe

In general, blood phenylalanine concentrations lower than 120 µM are considered normal levels (the average value being around 50 µM). Values higher than this concentration are associated with hyperphenylalaninemia, and plasma concentrations higher than 1000 µM are associated with the phenylketonuria disease. Therefore, the evaluation of the method was carried out for a range of l-Phe concentrations from 50 to 1000 µM, using the final conditions described in the previous section and following procedures set out in the experimental section “Synthesis of AuNMs stabilized by LAAO in the presence of l-Phe.” The results are shown in detail in ESM4. Figure S4.1A shows the absorption spectra in each case after 4 h of reaction, and Figure S4.1B shows the sigmoid adjustment of the absorbance at the maximum (550 nm) versus the corresponding l-Phe concentration. Thus, these results demonstrate the possibilities of the method for the detection of phenylalanine concentrations within the biological values of interest. However, the evaluation of the kinetics of the synthesis reaction for the various concentrations of phenylalanine tested (Figure S4.2) indicates that the formation of these nanoparticles begins at around 120 min and shows that at 4 h this absorbance still does not stabilize.

In order to improve the kinetics of the reaction, the use of the enzyme catalase was evaluated. This enzyme is able to catalyze the dismutation of the product of the H_2_O_2_ (Scheme 1), in water and oxygen. The section ESM5 shows the previous results that confirmed that the elimination of H_2_O_2_ present in the medium facilitates the regeneration of the enzyme, accelerating the rate of formation of AuNPs. Therefore, it was decided to carry out the final calibration of the method using catalase, in order to improve the sensitivity of the method and its kinetics.

**Fig. 2:**
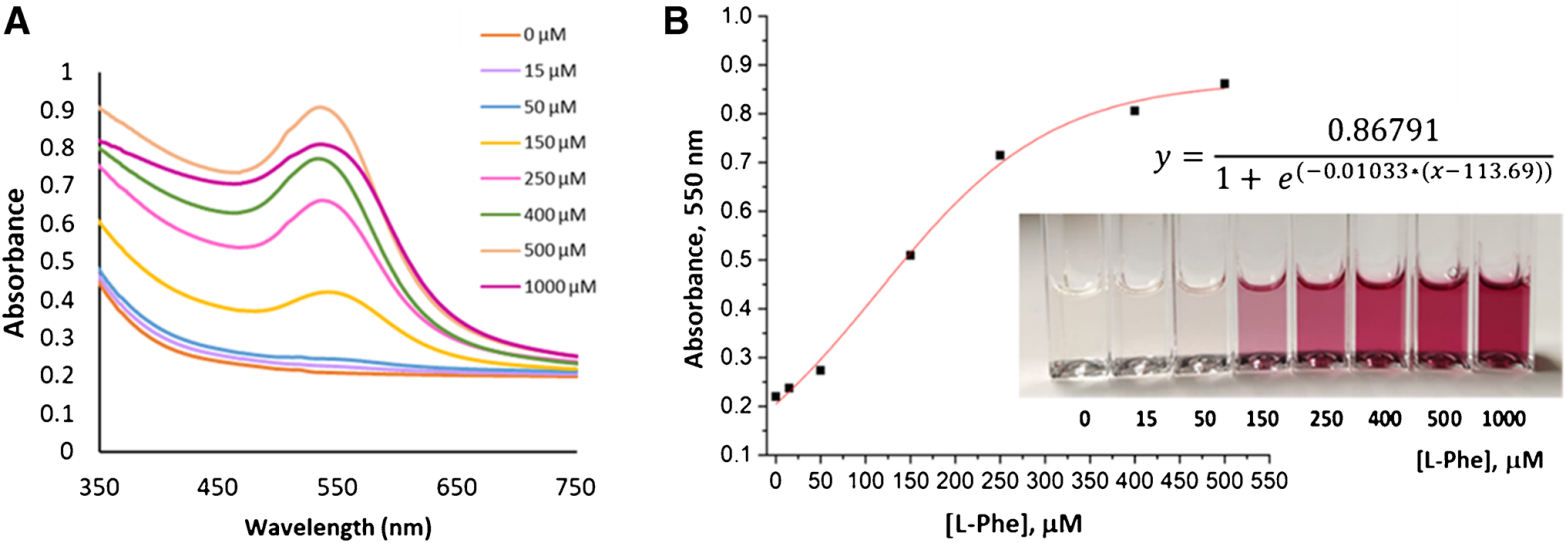
**A** Molecular absorption spectra obtained after 3 h of reaction for the different concentrations of l-Phe evaluated. **B** Sigmoid adjustment of the method for the determination of l-Phe in the range of 15 to 500 µM. The reaction was carried out in 0.1 M phosphate buffer pH 6.5 using 3.0 · 10^−6^ ML LAAO and 100 U catalase. After 20 min of reaction, 7 · 10^−4^ M Au(III) was added. Following 3 h at 37 °C, the absorption measurements were recorded

Figure 2A shows the absorption spectra after 3 h of reaction, where the saturation of the plasmon is observed at the highest concentration of 1000 µM. Figure 2B presents the sigmoid adjustment of the absorbance at the maximum (550 nm) versus the corresponding l-Phe concentration. The evaluation of the synthesis reaction kinetics for each concentration of l-Phe (Figure S4.3) shows that the beginning of the nanoparticle formation begins at around 30 min compared to 120 min shown by the method without catalase (Figure S4.2). It can also be observed that at 3 h the absorbance at 550 nm stabilizes, indicating the end of the reaction. In this way, the use of catalase allows the determination of lower concentrations of l-Phe and in a shorter time, assuming an increase in the sensitivity of the method compared to the same without catalase. Finally, the linearization of the sigmoid fit shown in ESM6 (Linearization of the sigmoid fit) allows obtaining the analytical quality parameters of the method with and without catalase. The use of catalase, in addition to affecting the kinetics by shortening the reaction time and decreasing the detection and quantification limit by a factor of about 6 (Table 1), produces highly competitive results for a rapid and colorimetric method of analysis compared to those obtained by the more complex chromatographic or bacterial inhibition methods used in practice.

**Table 1:** Equations and analytical figures of merit of the developed method with or without catalase

	Linear adjustment equation	Phe range (µM)	LOD (µM)	RSD (%)
Without catalase	*y* = 0.005*x* − 2.378	159–1000	59	5.2 (*n* = 3)
With catalase	*y* = 0.012*x* − 1.276	17–500	10	4.8 (*n* = 3)

#### Interference study

**Fig. 3:**
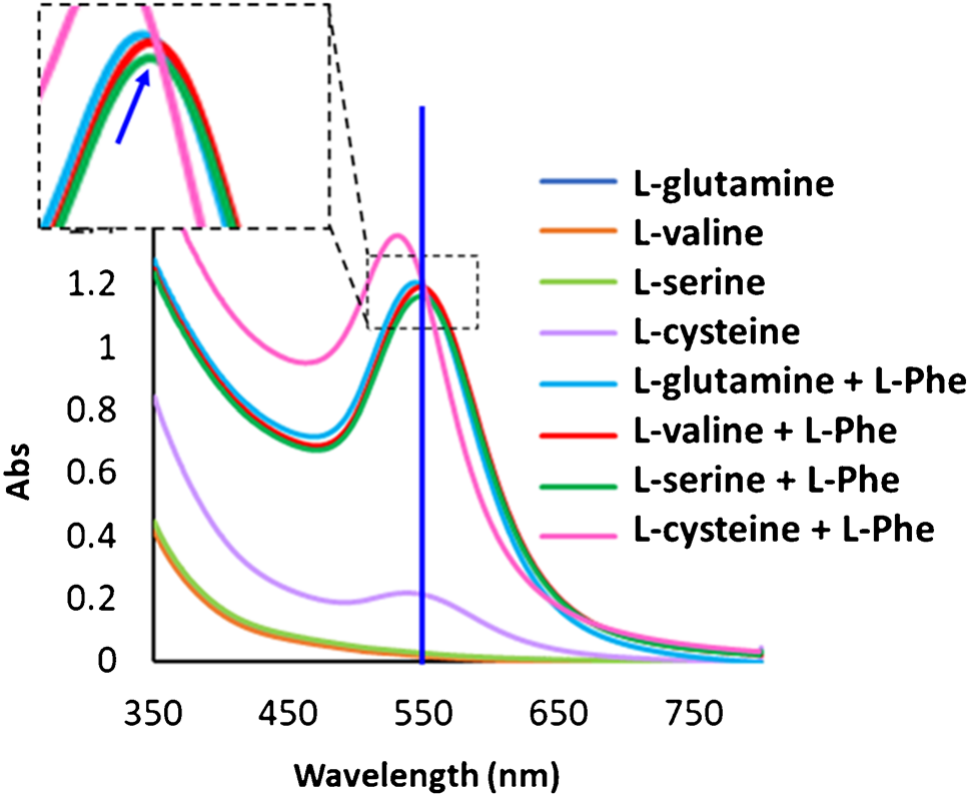
Molecular absorption spectra obtained in the various interference tests carried out applying the method developed for the determination of l-phenylalanine (800 μM). The reaction was carried out in 0.1 M phosphate buffer pH 6.5 using 3.0 · 10^−6^ M LAAO, and after 20 min of reaction, 7.0 · 10^−4^ M Au(III) was added. After 3 h at 37 °C, the absorption measurements were recorded

Finally, the effect of other l-amino acids present in blood plasma was studied as possible interferences in the detection of l-phenylalanine following the procedure described in experimental section “l-Phe quantification in human blood plasma” l-Glutamine and l-valine were selected for being the most abundant amino acids in plasma. The amino acid l-serine was selected due to its possible action as a reducing agent in the formation of nanoparticles (N. Jayaprakash et al. [34] described how the OH groups of serine are oxidized to C = O during the nitrate reduction reaction of silver (AgNO_3_) for the formation of silver nanoparticles). Finally, the amino acid l-cysteine was selected, due to the high affinity of the thiol groups for gold, which positions this amino acid as a possible interference. Figure 3 shows the absorption spectra obtained by applying the developed method under the optimized conditions both to the amino acids studied independently, and in the presence of the analyte, in order to evaluate whether they were capable of forming AuNPs. It is observed that none of them generate AuNPs, except for cysteine, due to the thiol-gold affinity. However, the spectra obtained for the mixtures with phenylalanine show, in all cases, the invariable plasmon peak at 550 nm, associated with the generation of AuNPs by the analyte. Only in the mixture containing cysteine and phenylalanine does the generated plasmon appear shifted toward lower wavelengths, 530 nm, as well as having a greater intensity. However, this does not disturb the final absorbance measured at 550 nm, which remains unchanged. Thus, this colorimetric method makes it possible to relate the amount of plasmonic nanoparticles (at 550 nm) with the concentrations of l-Phe with good selectivity.

#### l-Phe quantification in human blood plasma

To study the effect of the matrix and apply this methodology in plasma samples, known concentrations of l-Phe were added to a plasma solution following the procedure described in experimental section “l-Phe quantification in human blood plasma,” and analyzed to compare the sensitivity, LOD, and LOQ of the method in buffer. These results are shown in detail in ESM7 (“L-Phe quantification in human blood plasma”). Figure S7.1A shows the cuvettes together with the synthesis reaction kinetics (at 550 nm) for each concentration of l-Phe. Figure S7.1B shows the linear adjustment of the calibration. The analytical parameters of the method applied to human blood plasma samples were a LOD (3*standard deviation of the blank) of 22 µM and a RSD% (150 μM) 5.6% (*n* = 3). The recovery of two spiked samples gave a value of 110 ± 5% for a concentration of l-Phe 15 μM (*n* = 3) and 105 ± 3% for a concentration of l-Phe 350 μM (*n* = 3). The method shows a slightly higher LOD than for the method applied in buffer but remains competitive with respect to classical methods.

## Conclusions

This paper shows a simple analytical method, based on a specific biochemical reaction, which allows the determination of l-Phe directly, without sample treatment, and with good sensitivity and selectivity. It is important to highlight that the study described in this paper regarding the mechanism of the LAAO-l-Phe enzymatic reaction in the presence of Au(III) has allowed us to take a further step in the use of the redox properties of an enzymatic reaction in AuNP formation, showing that the mechanism of this kind of synthesis may vary depending on the enzyme used or due to the effect of some reaction products.

## Supplementary Information

Below is the link to the electronic supplementary material.

Supplementary file1 (DOCX 4.30 MB)
