# Supplementary material for: Gold nanoparticle formation as an indicator of enzymatic methods: colorimetric l-phenylalanine determination
Source: Anal Bioanal Chem. 2022 Jan 21;414(8):2641–9. doi: 10.1007/s00216-022-03900-3 (PMC8888390; doi:10.1007/s00216-022-03900-3)
Supplement: Supplementary file 1 — Supplementary file1 (DOCX 4.30 MB) [file 216_2022_3900_MOESM1_ESM.docx]

**GOLD NANOPARTICLE FORMATION AS AN INDICATOR OF ENZYMATIC METHODS: COLORIMETRIC L-PHENYLALANINE DETERMINATION**

**Electronic supplementary material**

**ESM1.- 3D structure of the LAAO from Crotalus rhodostoma**

**
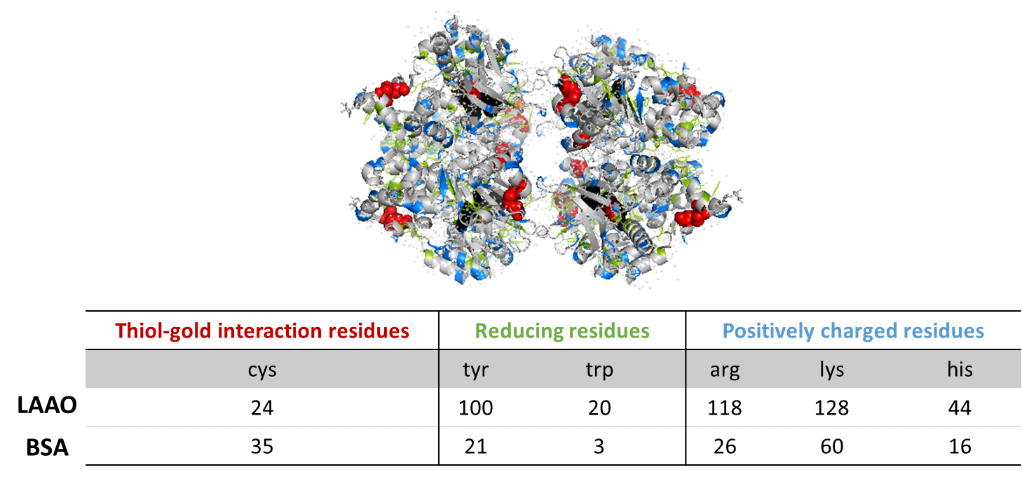
**

**Figure S1.1:** 3D structure of the LAAO from *Crotalus rhodostoma* (2iid.pdb) and content of key amino acid residues in the synthesis of AuNCs. In red cysteines (cys), in green reducing residues such as tyrosine (tyr) and tryptophan (trp), in blue positively charged amine residues like arginine (arg), lysine (lys) and histidine (his). In structure 3D, the 4 FADs of the dimeric glycoprotein LAAO are marked in black. The table shows the number of each of these amino acids in the LAAO structure and its comparison with those found in the BSA enzyme.

**ESM2.- Synthesis and characterization of AuNMs stabilized by LAAO in the absence of L-Phe**


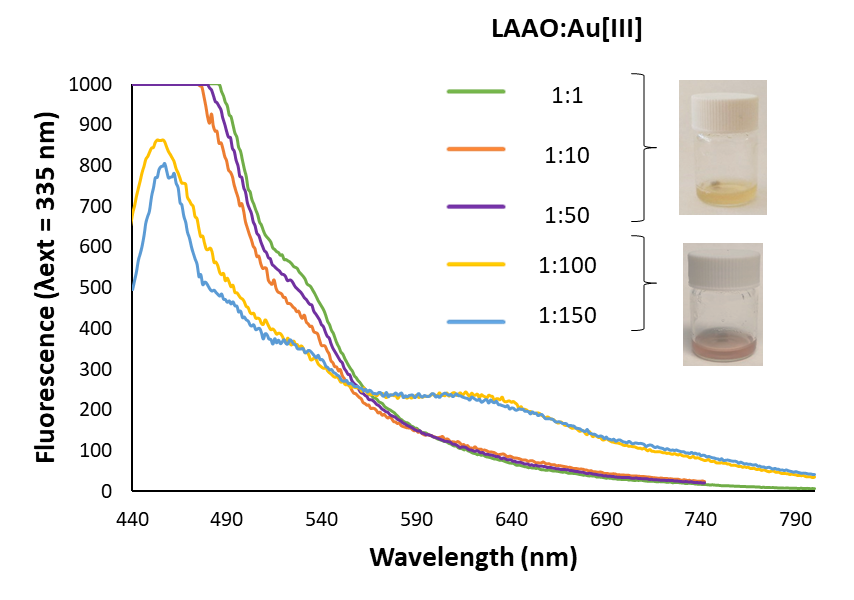


**Figure S2.1:** Emission spectra of the various syntheses performed with various molar ratios LAAO:Au(III) at a fixed enzyme concentration of 4.8·10^-5^ M. Excitation is performed at 335 nm and a single emission maximum is observed at 620 nm for higher concentrations of gold.


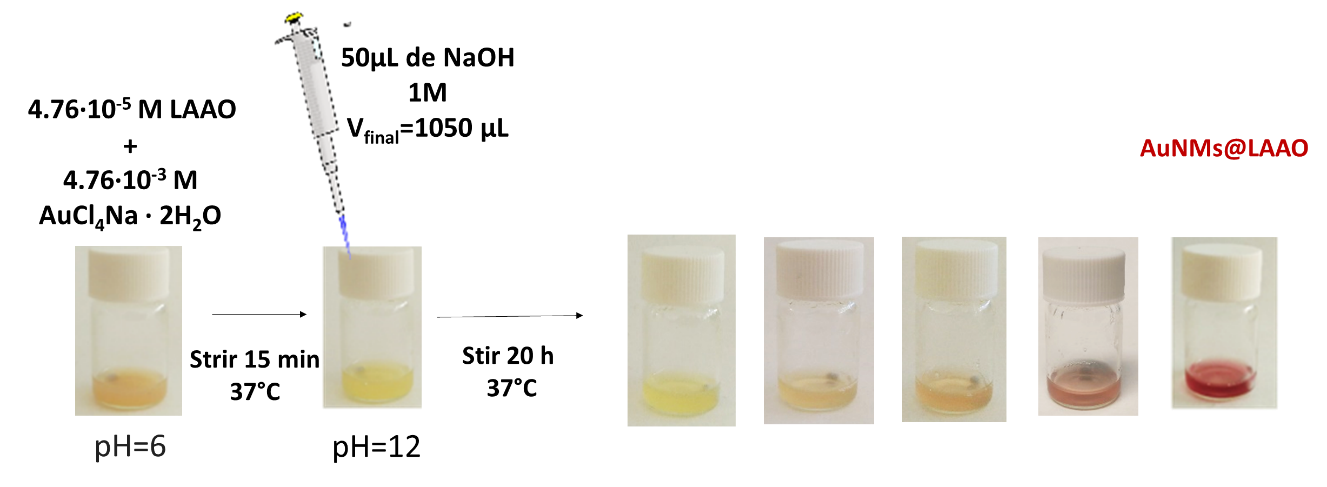


**4.8**

**4.8**

**Figure S2.2:** Final protocol for the synthesis of AuNMs@LAAO and appearance of the solutions in each of the reaction times of the synthesis at pH 12.


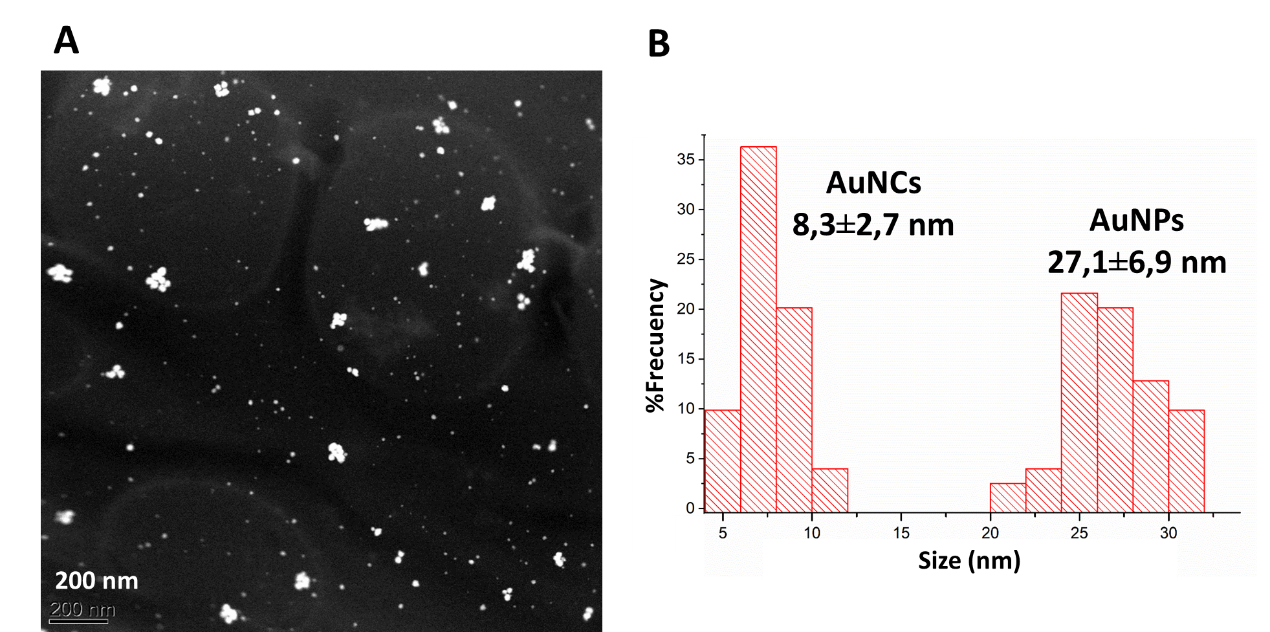


**Figure S2.3:** A) STEM image of the synthesized AuNMs@LAAO. B) Corresponding histogram representing the particle size distribution.


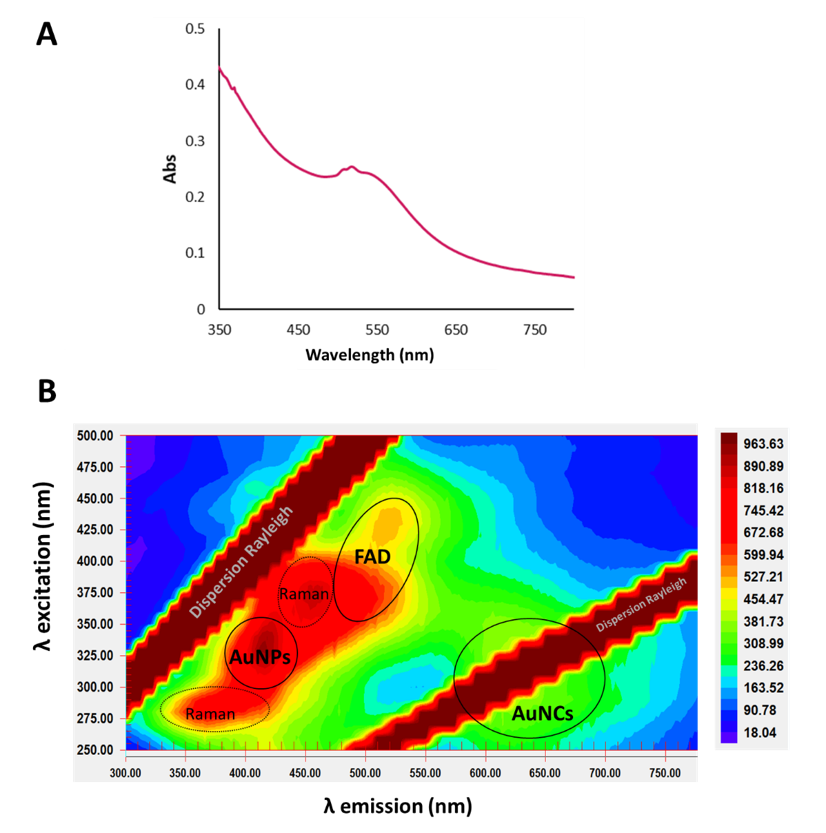


**Figure S2.4:** A) absorption spectrum of the final synthesis B) 3D fluorescence spectrum of the synthesis performing an excitation scan from 250 to 500 nm, measuring the emission from 300 to 800 nm; the intensity corresponding to the different colors is shown on the right.


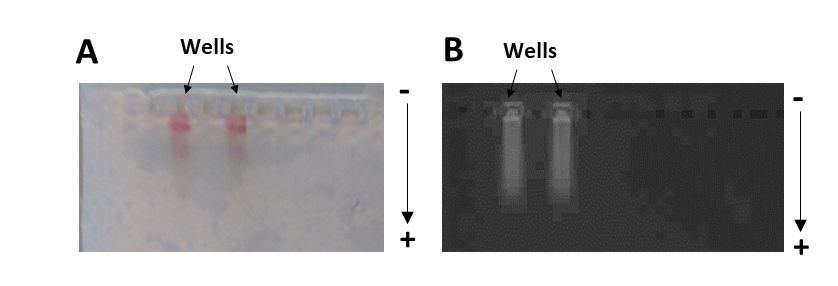


**Figure S2.5:** A) Appearance of the agarose gel after electrophoresis for the separation of AuNMs@LAAO, where the reddish AuNPs (larger population sizes) are observed within the gel generating a reddish band, somewhat more intense just at the outlet of the well. B) Appearance of this same gel in a UV lamp, where the fluorescence bands generated by these red AuNPs can be observed and also inside the well.


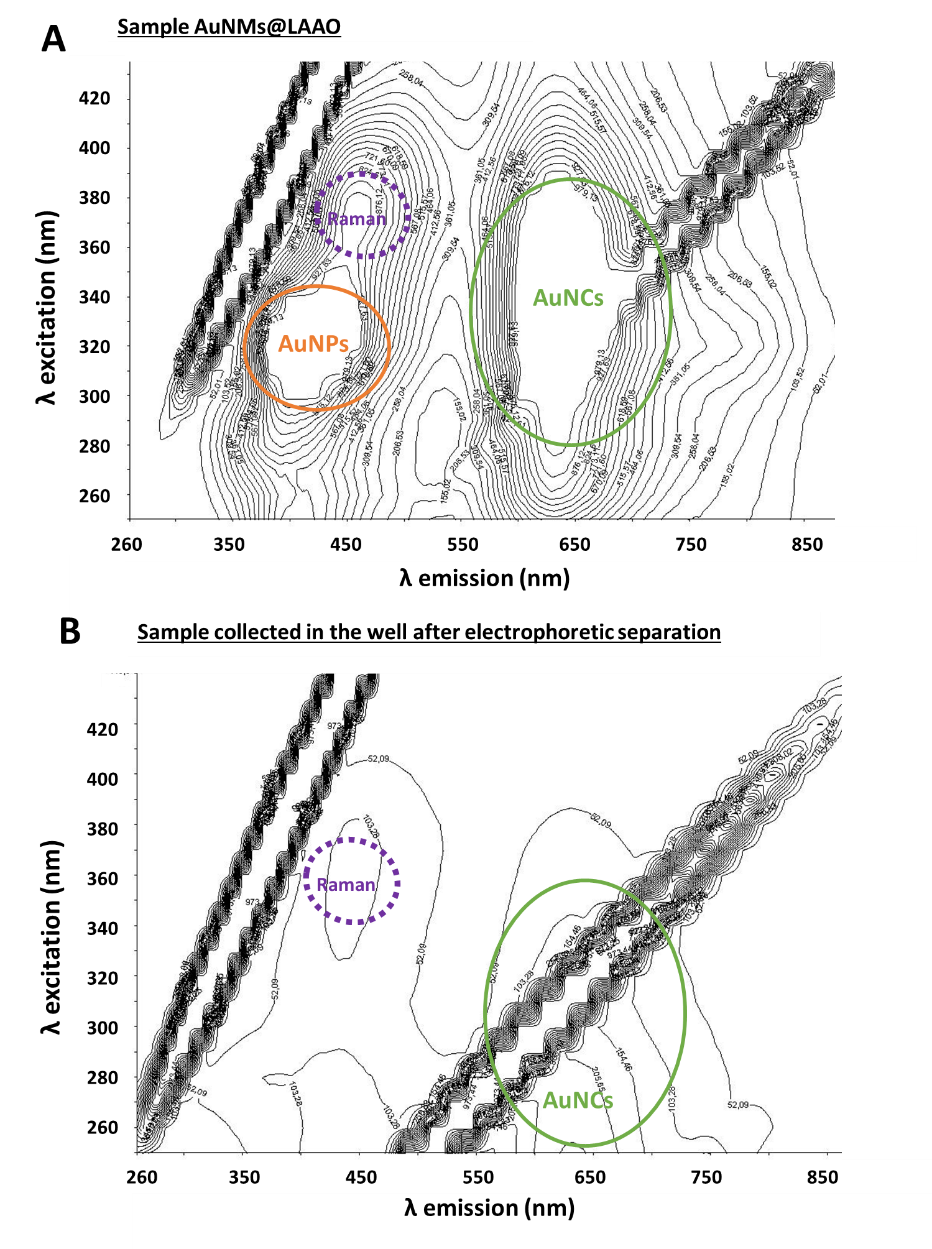


**Figure S2.6:** 3D fluorescence spectra performing an excitation scan from 250 to 450 nm, measuring the emission from 260 to 850 nm of samples A) Sample AuNMs@LAAO original B) sample collected in the well after electrophoretic separation. Comparison of both 3D spectra shows, after separation, the disappearance of the maximum fluorescence of 420 nm, associated with the larger AuNPs with plasmon, which enter the gel (for charge effect) and show the elongated band of fluorescence.


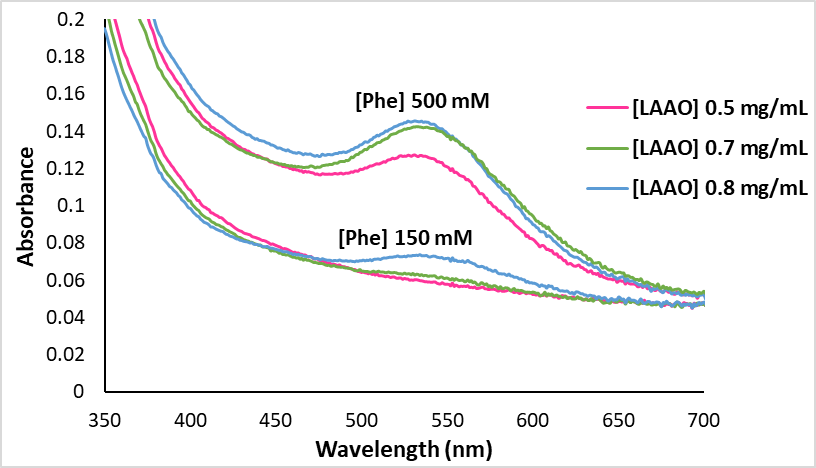

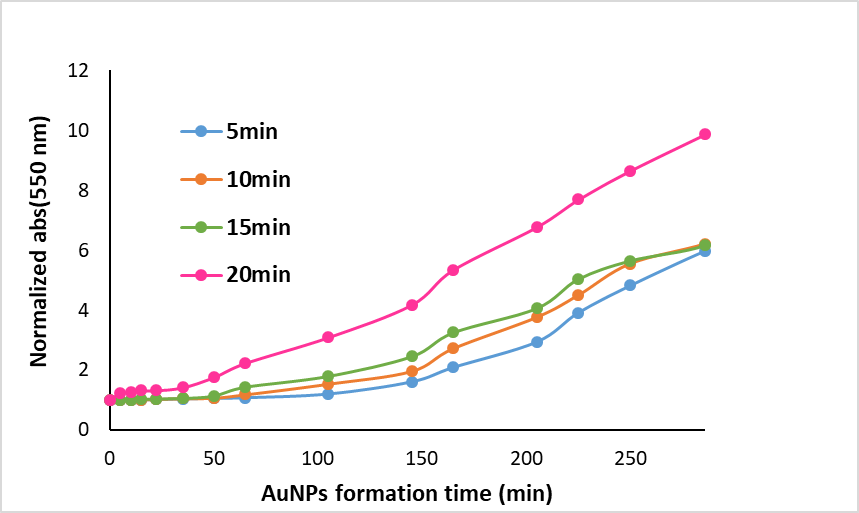
**ESM3.- Synthesis optimization of AuNMs stabilized by LAAO in the presence of L-Phe**


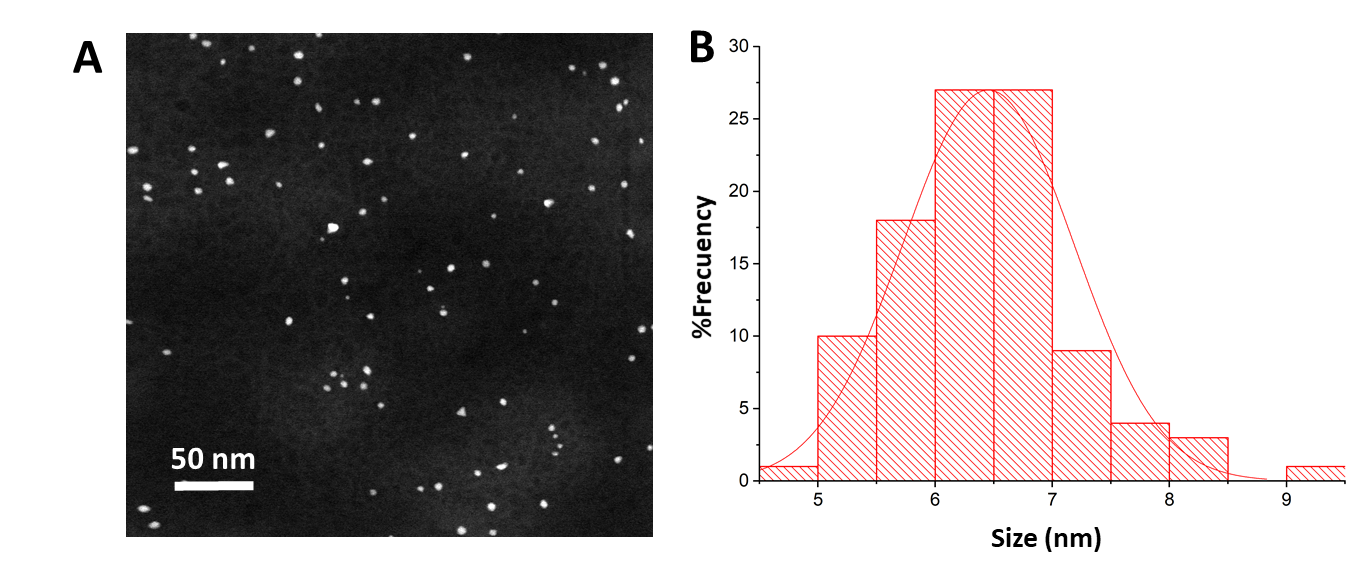


**Figure S3.1:** AuNPs synthesized in 0.1M phosphate buffer pH 6.5 using 0.8 mg/mL LAAO and 500 µM Phe, after 20 minutes of reaction, 0.7 mM Au (III) was added. After 3 h at 37 ° C, A) STEM image of the synthesized nanoparticles was obtained. B) Corresponding histogram representing the particle size distribution.


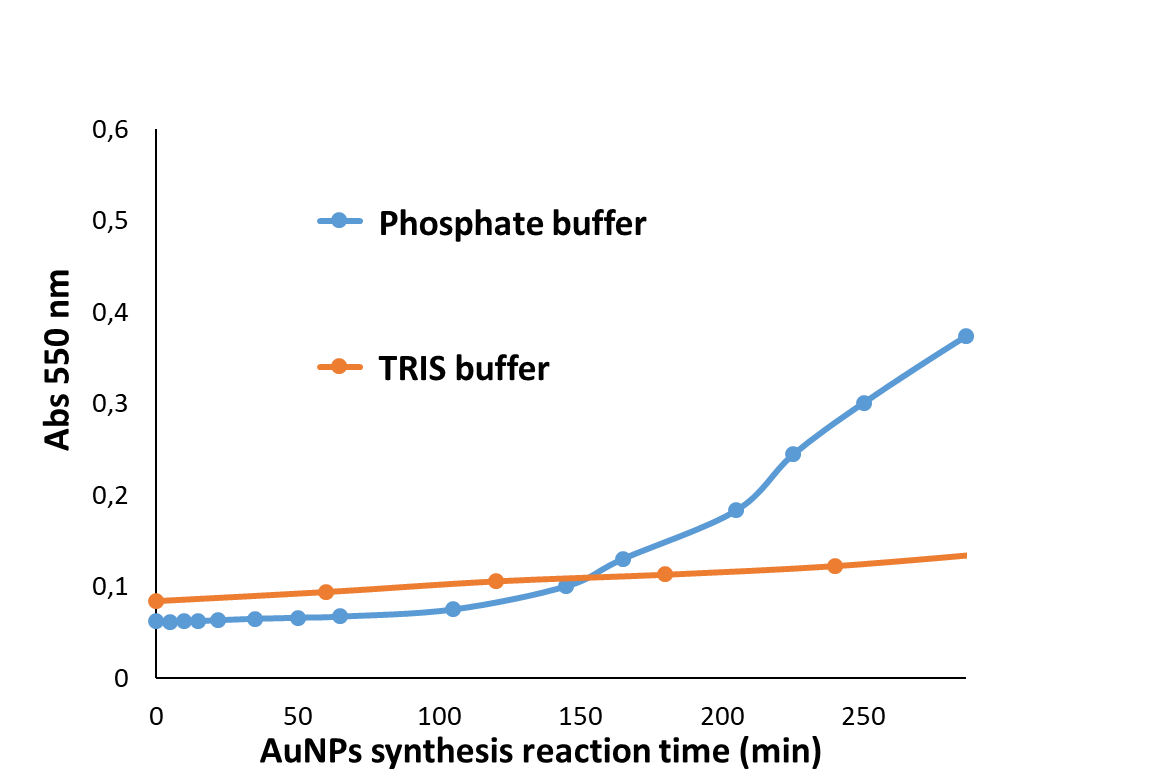


**Figure S3.2:** Kinetics of the synthesis reaction of AuNPs in 0.1 M pH 6.5 phosphate buffer and 0.1 M pH 6.5 TRIS buffer, under the conditions: LAAO 3.0·10^-6^ M; L-Phe 500 μM; LAAO-L-Phe reaction time 5 minutes; Au (III) 7·10^-4^ M.

**
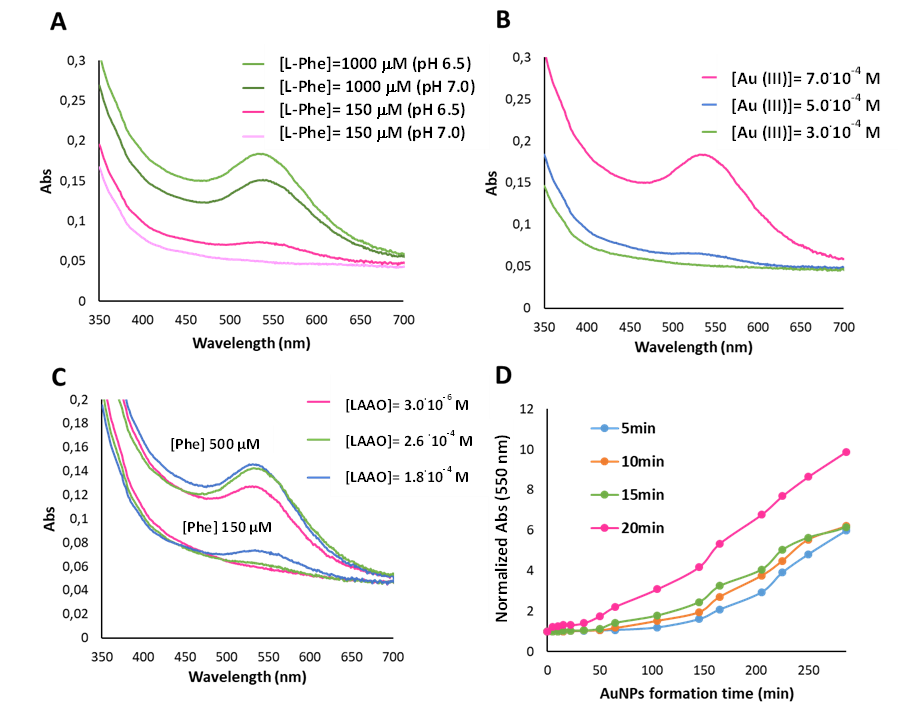
**

**Figure S3.3:** Synthesis optimization study: A) pH: [LAAO] = 3.0·10^-6^ M, [L-Phe]=150 µM and 1000 µM; [Au (III)] = 7.0·10^-4^ M; B) Au (III) concentration: 0.1M pH 6.5 (phosphate), [LAAO] = 3.0·10^-6^ M and [L-Phe]=1000 µM; C) LAAO concentration: 0.1M pH 6.5 (phosphate), L-Phe]=150 µM and 500 µM, [Au (III)] = 7.0·10^-4^ M. For studies A, B and C, Au (III) is added after 5 minutes of LAAO-L-Phe reaction. D) LAAO-L-Phe reaction time before gold addition: [LAAO] = 3.0·10^-6^ M in 0.1M phosphate buffer pH 6.5; [L-Phe]=500 µM, [Au (III)] = 7.0·10^-4^ M.


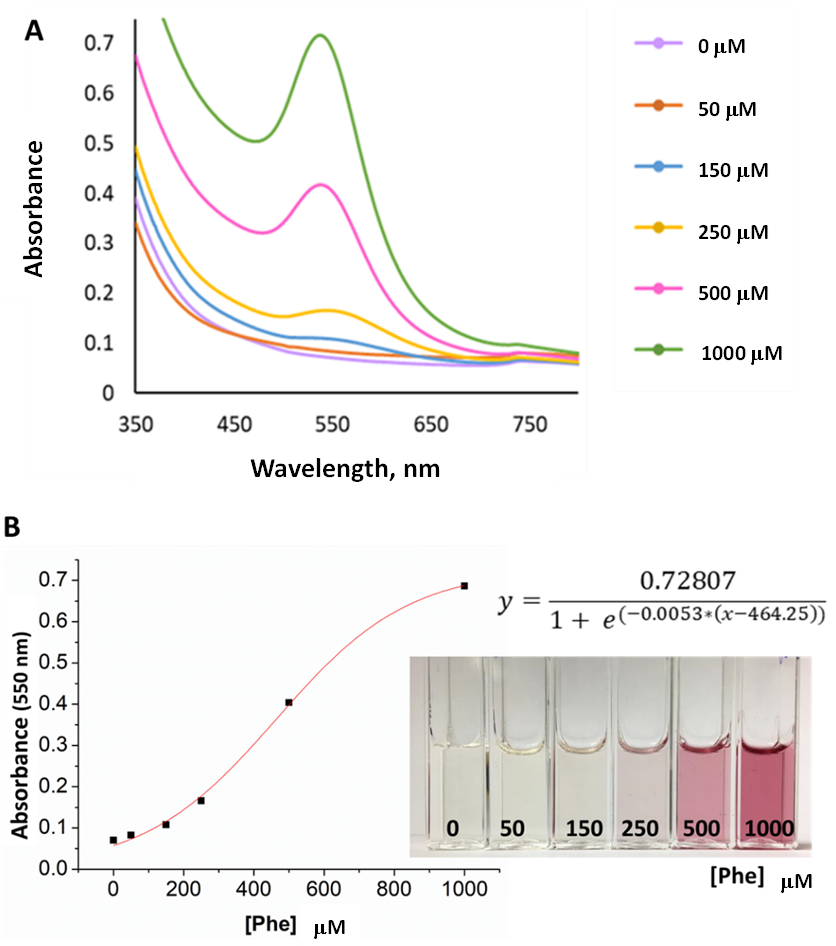
**ESM4.- Calibration study of the method for the determination of L-Phe**

**Figure S4.1:** A) Molecular absorption spectra obtained after 4 hours of reaction for the different concentrations of L-Phe evaluated. B) Sigmoid adjustment of the method for the determination of L-phenylalanine in the range of 50 to 1000 µM. The reaction was carried out in 0.1M phosphate buffer pH 6.5 using [LAAO] = 3.0·10^-6^ M and after 20 minutes of reaction, [Au (III)] = 7.0·10^-4^ M was added. After 4h at 37° C, the absorption measurements of the cuvettes shown were recorded.


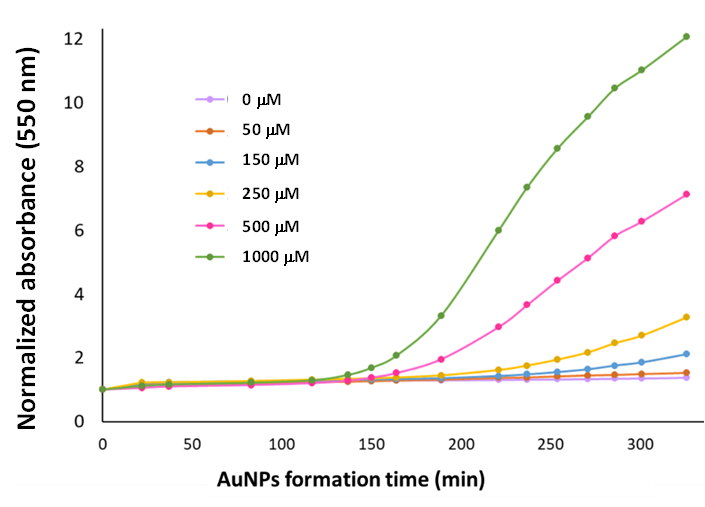


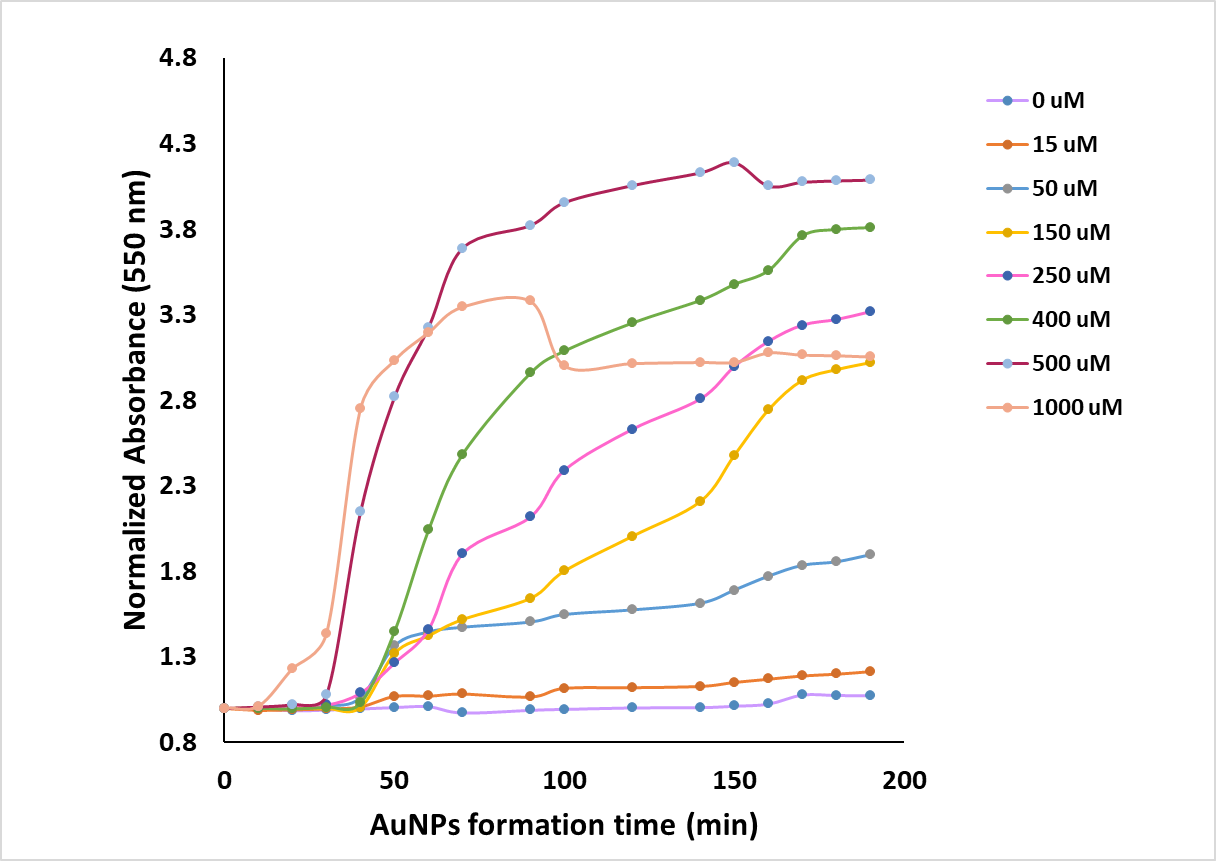


**Figure S4.2:** Variation of absorbance at 550 nm during the 4h of synthesis reaction of the AuNPs for the various concentrations of phenylalanine evaluated. The reaction was carried out at 37° C in 0.1M phosphate buffer pH 6.5 using [LAAO] = 3.0·10^-6^ M and after 20 minutes of reaction, [Au (III)] = 7.0·10^-4^ M was added.

**Figure S4.3:** Variation of absorbance at 550 nm during the 3 hours of synthesis reaction of the AuNPs for the various concentrations of phenylalanine evaluated. The reaction was carried out at 37° C in 0.1M phosphate buffer pH 6.5 using [LAAO] = 3.0·10^-6^ M and 100 U of catalase. After 20 minutes of reaction, Au (III)] = 7.0·10^-4^ M was added.


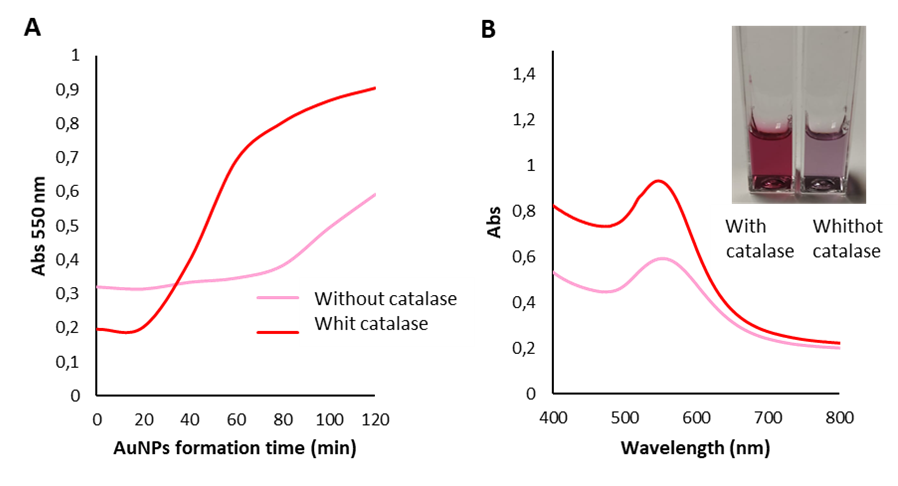
**ESM5.- Study of reaction kinetics using catalase**


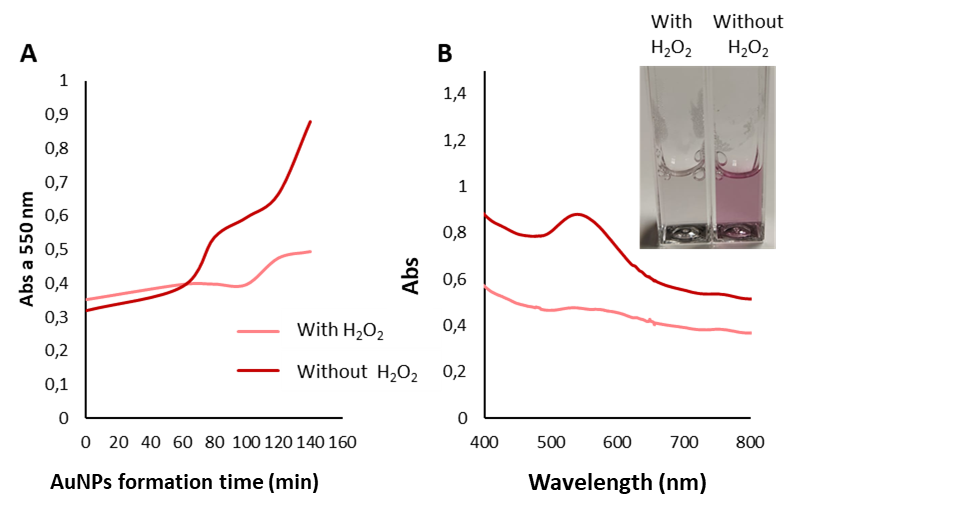


**Figure S5.1:** Variation of the absorbance at 550 nm during the synthesis of the AuNPs for the determination of L-phenylalanine 500 µM with the optimized reaction conditions, in the presence and absence of catalase (100U total). B) Molecular absorption spectra and appearance of the cuvettes after 2h of reaction.

**Figure S5.2:** A) Effect of the addition of 500 µM H_2_O_2_ on the variation of absorbance at 550 nm during the synthesis of AuNPs for the determination of 500 µM L-phenylalanine with the optimized reaction conditions and in the absence of catalase. B) Molecular absorption spectra and appearance of the cuvettes after 2 hours and a half of reaction.

**ESM6.- Linearization of the sigmoid fit**

The developed method presents a sigmoid response calibration, due to this type of curve is difficult to handle, an adequate interpretation of the coefficients that appear in it would facilitate its linearization. Of the different mathematical models that generate sigmoid curves, the so-called “exponential growth with carrying capacity” (logistic growth) is applied to the growth of populations (such as bacteria) when there is limited resources (such as food); in this case, the limiting resource would be the analyte concentration. The generic equation of this model is:

$\frac{dy}{dx}=k\left( \frac{y_{max}-y}{y_{max}} \right)y$ (**Eq. 1**)

Where *y* is the dependent variable and *x* is the independent one. The y_max_ value is the “load capacity” (the maximum value that *y* can reach, that is, the limiting value) and *k* is the maximum growth rate, a parameter that is characteristic of the system. The application of our system to the equation (Eq.1) results in the following way:

$\frac{dAbs}{dc}=k\left( \frac{{Abs}_{max}-Abs}{{Abs}_{max}} \right)Abs$ (**Eq. 2**)

Integrating the (Eq.2):

$Ln\left( \frac{Abs}{{Abs}_{max}-Abs} \right)=KC+ Ln\left( \frac{{Abs}_{0}}{K-{Abs}_{0}} \right)$ (**Eq. 3**)

Where Abs_max_ is the maximum absorbance obtained, Abs_0_ is the absorbance in the absence of analyte and k depends on the chemical system (on the reaction and its conditions). If we call C_m_ to the concentration of analyte that gives a value of Abs equivalent to half the maximum (Abs_max_/2), the equation (Eq.3) becomes:

$Ln\left( \frac{Abs}{{Abs}_{max}-Abs} \right)=KC-KC_{m}=K\left( C-C_{m} \right)$ (**Eq.4**)

Therefore, it is possible to establish a linear relationship between a function of Abs and C (as long as Absmax is known). As might be expected, if equation (Eq. 4) is rearranged, it remains:

$$Abs=\frac{{Abs}_{max}}{1+e^{-K\left( C-C_{m} \right)}} (\mathbf{Eq. 5})$$

This equation is of the same type as those obtained in the sigmoid adjustments for the method without catalase (Eq. 6) and with catalase (Eq. 7):

$$Abs=\frac{0,728}{1+e^{-0,0053\left( C-464,2 \right)}} (\mathbf{Eq. 6})$$

$$Abs=\frac{0,868}{1+e^{-0,0103\left( C-113,7 \right)}} (\mathbf{Eq. 7})$$

By applying the inverse operation to these equations to those performed to go from (Eq. 4) to (Eq. 5), the logarithmic equations are obtained respectively for the method without catalase (Eq. 8) and with catalase (Eq. 9):

$$Ln\left( \frac{Abs}{0,728-Abs} \right)=0,0053C-2,460 (\mathbf{Eq. 8})$$

$$Ln\left( \frac{Abs}{0,868-Abs} \right)=0,0103C-1,171 (\mathbf{Eq. 9})$$

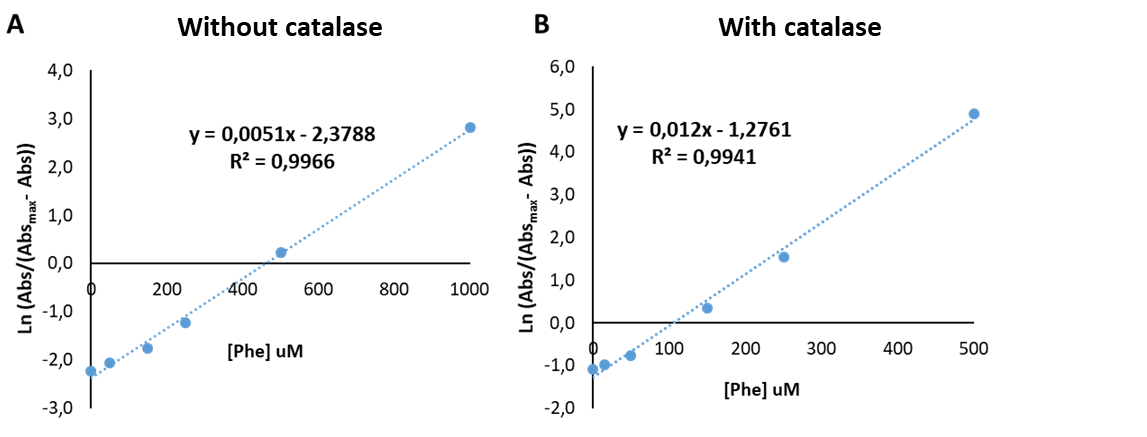
From the Abs data obtained in each calibration and representing Ln (Abs/(Abs_max_-Abs)) versus the concentration of L-Phe in each case, the graphs shown in Figure S5.1 are obtained for the method with and without catalase along with their equations. It is observed that these equations are practically the same as those obtained from the sigmoid adjustments (Eq. 8) and (Eq. 9), thus confirming the linear relationship between the logarithmic function of Abs and the concentration of L-Phe.

**Figure S6.1:** Linear adjustment of the calibrations of the developed method, the reaction was carried out in both cases at 37 °C in 0.1 M phosphate buffer pH 6.5 using LAAO 0.8 mg/mL. After 20 minutes of reaction, 0.7 mM Au (III) was added. This reaction was studied A) without catalase and with B) catalase 100 U total.

From these equations, the detection and quantification limits were obtained in each case as analytical quality parameters of the method. In this case, the detection limit is the analyte concentration that would give an absorbance:

$${Abs}_{LD}= {Abs}_{blank \left( interpolate \right)}+3s_{blank} (\mathbf{Eq. 10})$$

To obtain this concentration, first the Abs_blank_ must be calculated (interpolated), for this, in the equation (Eq. 3) C is replaced by zero and the Abs.

$${Abs}_{blanco}= {Abs}_{max}\left( \frac{e^{-KC_{m}}}{1+e^{-KC_{m}}} \right) \left( \mathbf{Eq. 11} \right)$$

To this absorbance of blank (Eq. 11) 3s_blank_ is added and the value is interpolated in the equation (Eq. 3), that is:

$$Ln\left( \frac{{Abs}_{max}\left( \frac{e^{-KC_{m}}}{1+e^{-KC_{m}}} \right) +3s_{bl}}{{Abs}_{max}-\left( {Abs}_{max}\left( \frac{e^{-KC_{m}}}{1+e^{-KC_{m}}} \right) +3s_{bl} \right)} \right)=KC-KC_{m} (\mathbf{Eq. 12})$$

Solving for C from equation (Eq. 12) the limit of detection is obtained. To calculate the limit of quantification, only the value 3 is replaced in this equation by 10.


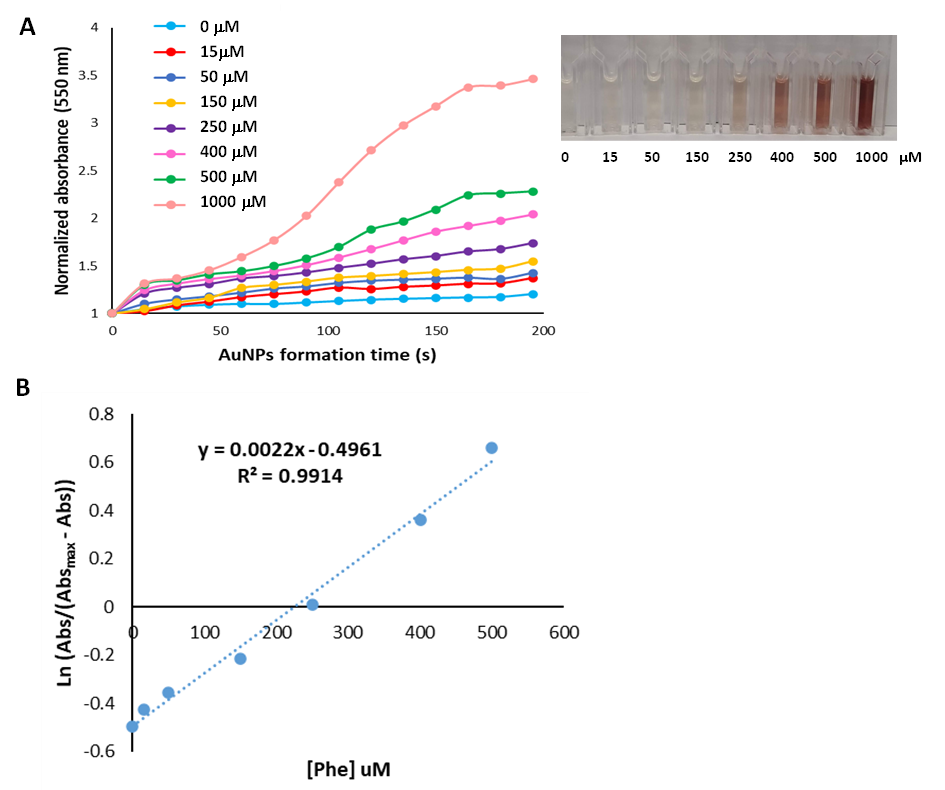
**ESM6.-** **L-Phe quantification in human blood plasma.**

**Figure S6.1:** A) Variation of absorbance at 550 nm during the 3.5 hours of synthesis reaction of the AuNPs for the various concentrations of phenylalanine evaluated in human blood plasma. The reaction was carried out at 37° C using a 1/2.5 plasma dilution in 0.1M phosphate buffer pH 6.5, [LAAO] = 3.0·10^-6^ M and 100 U of catalase. After 20 minutes of reaction, Au (III)] = 7.0.0·10^-4^ M was added. Then, the absorption measurements of the cuvettes shown were recorded. B) Linear adjustment.
